# Supplementary material for: Electronic nature of charge density wave and electron-phonon coupling in kagome superconductor KV3Sb5
Source: Nat Commun. 2022 Jan 12;13:273. doi: 10.1038/s41467-021-27946-6 (PMC8755796; doi:10.1038/s41467-021-27946-6)
Supplement: Supplementary file 1 — Supplementary Information [file 41467_2021_27946_MOESM1_ESM.pdf]

Supplementary Materials for

Electronic Nature of Charge Density Wave and Electron-Phonon Coupling in Kagome Superconductor KV_3Sb_5

Hailan Luo[1,2,6], Qiang Gao[1,6], Hongxiong Liu[1,2,6], Yuhao Gu[1,6], Dingsong Wu[1,2], Changjiang Yi[1], Junjie Jia[1,2], Shilong Wu[1], Xiangyu Luo[1,2], Yu Xu[1], Lin Zhao[1], Qingyan Wang[1], Hanqing Mao[1], Guodong Liu[1,2], Zhihai Zhu[1], Youguo Shi[1*], Kun Jiang[1*], Jiangping Hu[1,2], Zuyan Xu[3] and X. J. Zhou[1,2,4,5*]

[1]*Beijing National Laboratory for Condensed Matter Physics, Institute of Physics, Chinese Academy of Sciences, Beijing 100190, China*

[2]*University of Chinese Academy of Sciences, Beijing 100049, China*

[3]*Technical Institute of Physics and Chemistry, Chinese Academy of Sciences, Beijing, China*

[4]*Songshan Lake Materials Laboratory, Dongguan 523808, China*

[5]*Beijing Academy of Quantum Information Sciences, Beijing 100193, China*

[6]*These people contributed equally to the present work.*

[*]*Corresponding authors: ygshi@iphy.ac.cn, jiangkun@iphy.ac.cn and xjzhou@iphy.ac.cn.*

(Dated: December 10, 2021)

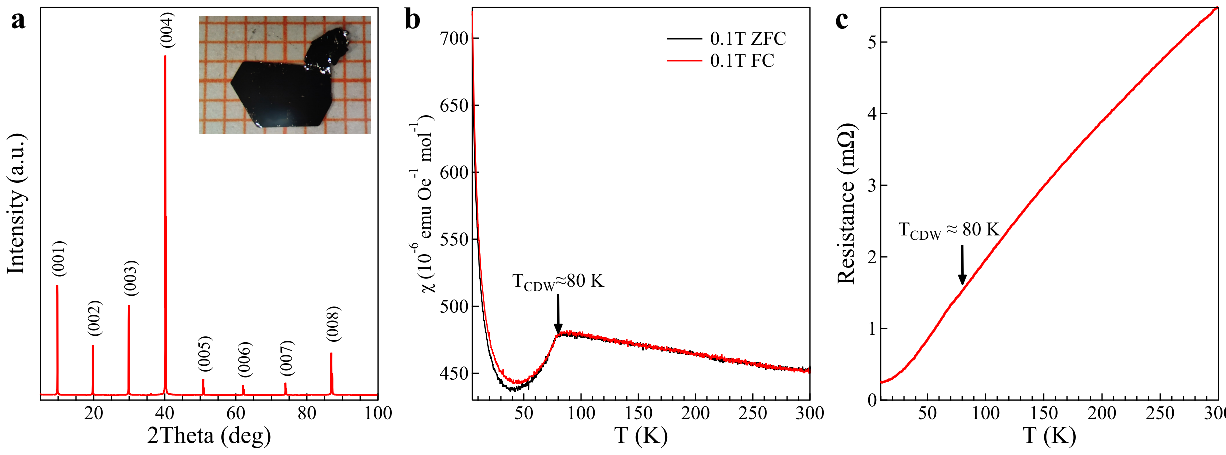

Supplementary Fig. 1: **Sample characterization and physical property measurements of KV_3Sb_5.** (a) XRD pattern of a KV_3Sb_5 single crystal. All the observed peaks can be indexed to (00L). The upper-right inset shows a photo of a typical KV_3Sb_5 single crystal on a paper with 1×1 mm^2 grids. (b) Temperature-dependent magnetic susceptibility of KV_3Sb_5 single crystal measured by both field cooled (FC) and zero field cooled (ZFC) modes. The CDW transition occurs at $\sim$80K that is marked by an arrow. (c) Temperature-dependent in-plane resistance of KV_3Sb_5 single crystal. The CDW transition at $\sim$80K is marked by an arrow.

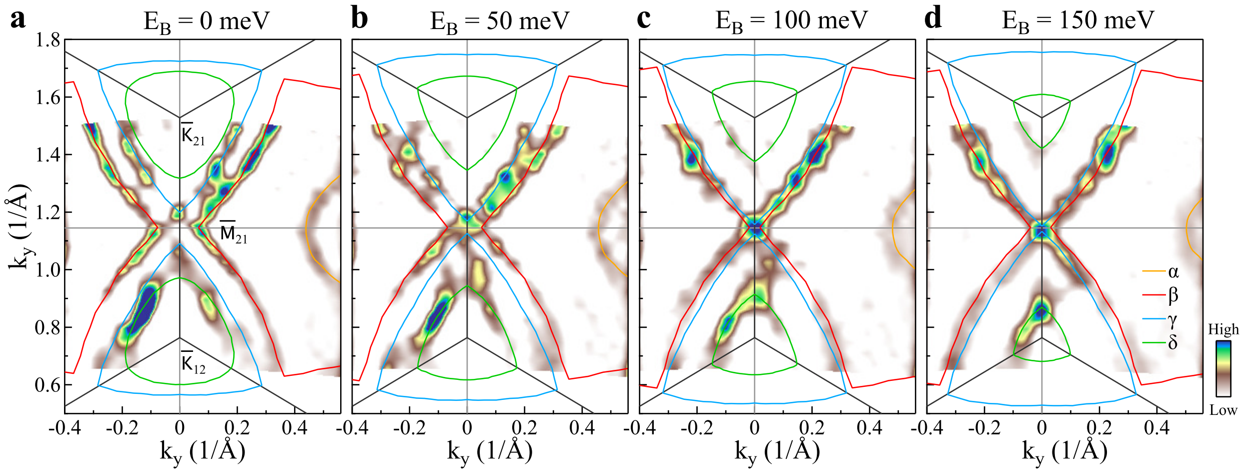

Supplementary Fig. 2: **Constant energy contours of KV_3Sb_5 measured at 5 K.** (a-d) Constant energy contours at different binding energies of 0 (a), 50 meV (b), 100 meV (c) and 150 meV (d), respectively. These are second derivative images with respect to momentum obtained from the same measurement as shown in Fig. 4l. The observed four Fermi surface sheets α, β, γ and δ are marked. With increasing binding energy, the α Fermi surface around $\bar{\Gamma}$ decreases in its size, indicating it is electron-like. The size of the hexagonal β Fermi surface around $\bar{\Gamma}$ increases with increasing binding energy and it is hole-like. The γ and δ Fermi surface show distinct matrix element effects. The γ Fermi surface is clearly observed around $\bar{K}_{21}$ but it is weak around $\bar{K}_{12}$. Its size increases with the increasing binding energy, indicating it is hole-like. On the other hand, the δ Fermi surface is clearly observed around $\bar{K}_{12}$ but it is weak around $\bar{K}_{21}$. Its size decreases with the increasing binding energy, indicating it is electron-like.

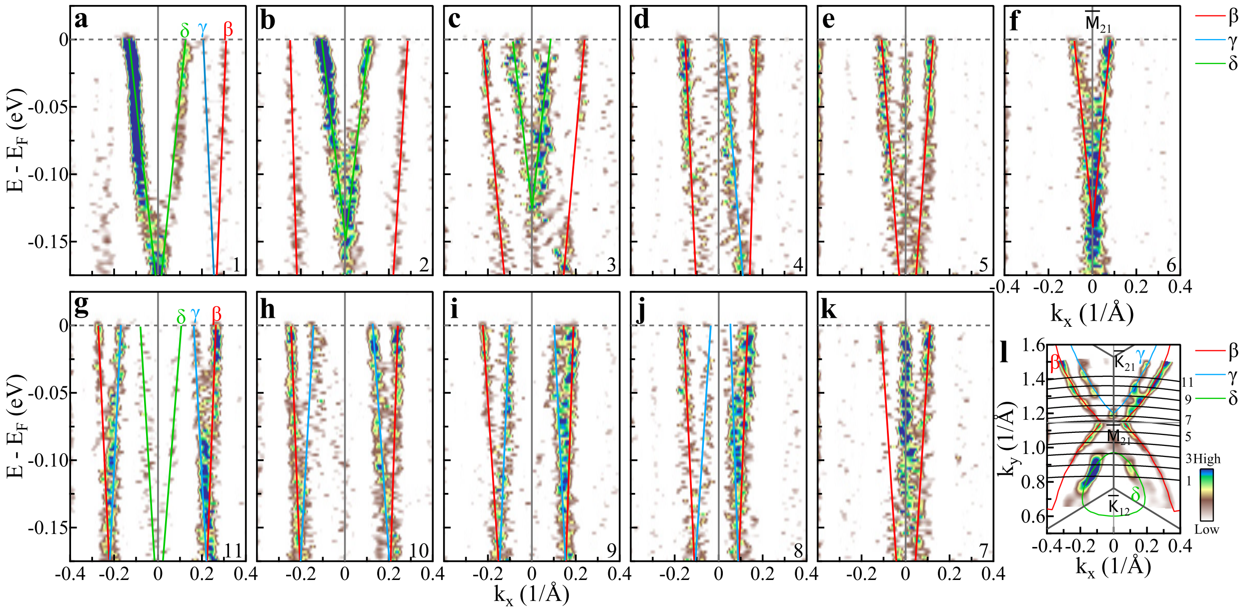

Supplementary Fig. 3: **Momentum-dependent band structure of KV_3Sb_5 measured at 5 K.** (a-k) Band structures measured along different momentum cuts. The locations of these momentum cuts 1-11 are marked in (l). These are the second derivative images with respect to momentum. Three kind of bands α, β and γ are observed which correspond to the three Fermi surface sheets in (l) and Fig. S2. The β band is clearly observed along all the momentum cuts. The δ band is strong in cuts 1, 2 and 3 in the momentum space around $\bar{K}_{12}$ but weak in the momentum space around $\bar{K}_{21}$. On the other hand, the γ band is obvious in cuts 8, 9, 10 and 11 in the momentum space around $\bar{K}_{21}$ but weak in the momentum space around $\bar{K}_{12}$. For some momentum cuts like 1 and 11, both the γ and δ bands can be observed simultaneously. (l) High-resolution Fermi surface mapping of KV_3Sb_5 at 5 K. It is a second derivaitve image with respect to momentum. The observed three Fermi surface sheets α, β and γ are marked.

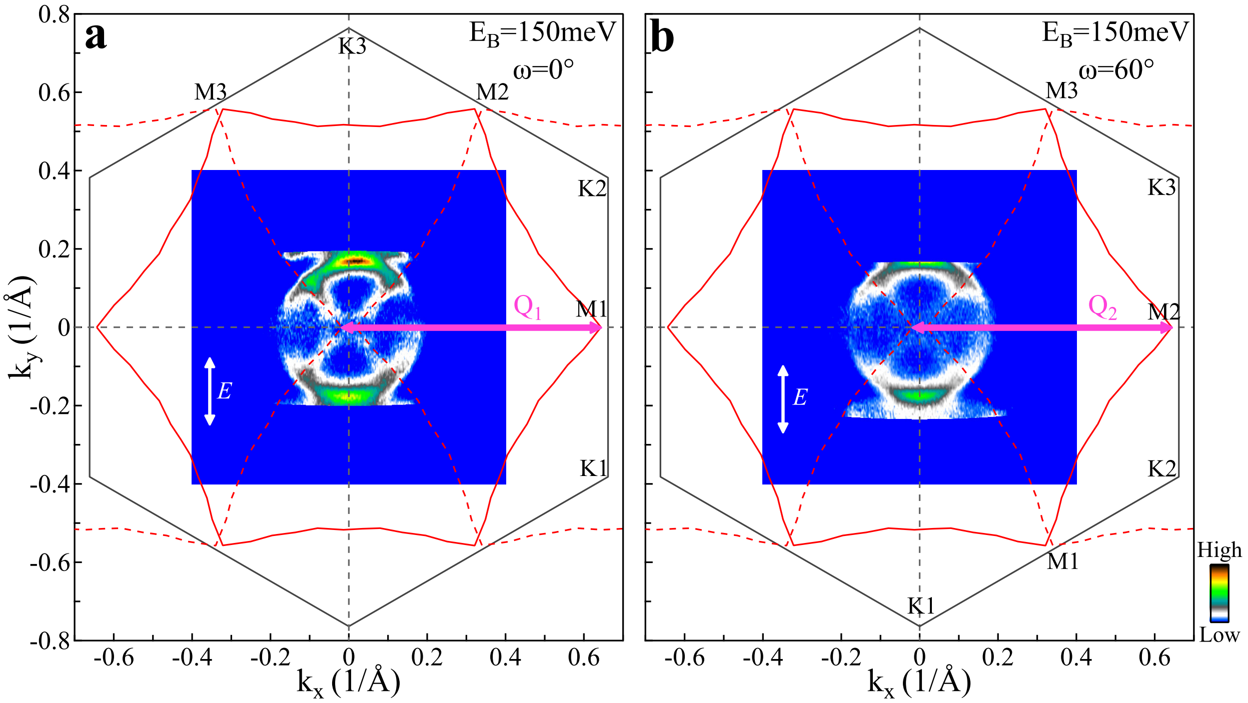

Supplementary Fig. 4: **Constant energy contours at $E_B = 150\,\text{meV}$ of KV_3Sb_5 measured by laser-ARPES at 20 K.** The sample in (b) is rotated clockwise by 60° with respect to the one in (a) while keeping all the other measurement conditions the same. Before the sample rotation, the folded bands are mainly from Q_1 wavevector (a). After the sample is rotated clockwise by 60°, the observed folded bands are mainly from Q_2 wavevector (b). This measurement demonstrates that different wavevectors can be observed under different measurement geometries.

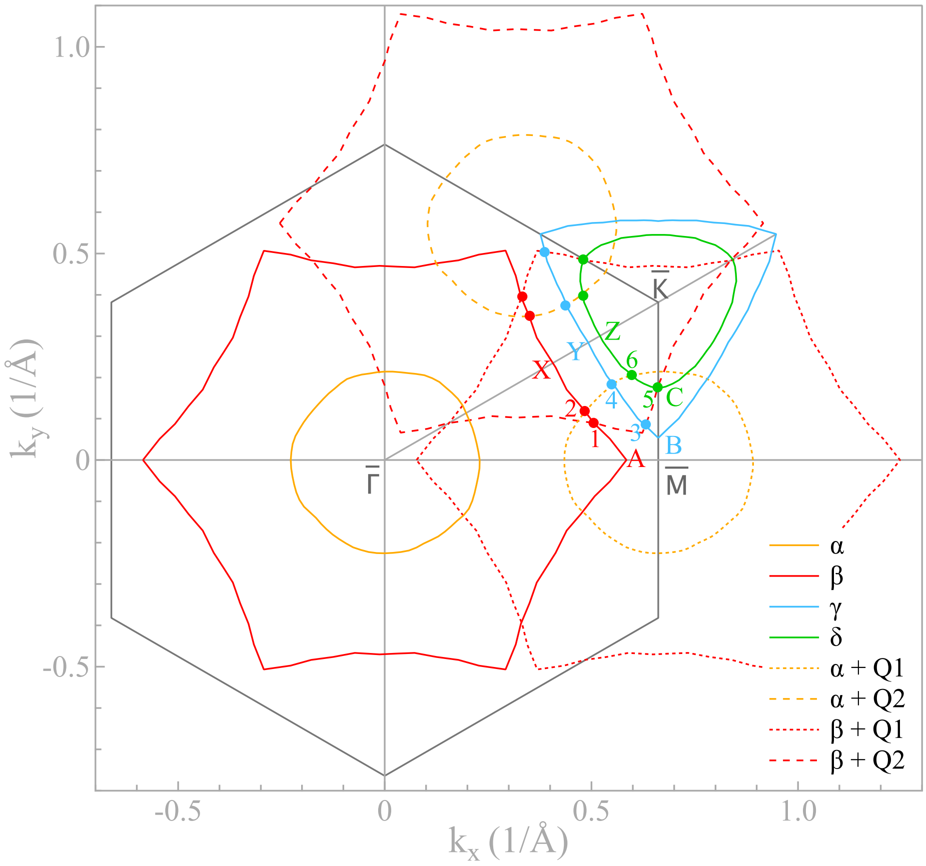

Supplementary Fig. 5: **The expected CDW gap openings in KV_3Sb_5.** The original Fermi surface α, β, γ and δ are plotted in solid lines while the folded Fermi surface sheets are plotted in dashed lines. The crossing points between the original and the folded Fermi surface sheets are marked by solid circles and numbers. The CDW gap is expected to open at the crossing points between the original and folded Fermi surfaces. For the β Fermi surface, two crossing points (1 and 2) are formed near the middle between A and X points. This would give a CDW gap maximum near the middle between A and X points and gap minimums at A and X points. This is consistent with our measured results in Fig. 4. For the γ Fermi surface, the observed crossing point 3 is close to the Fermi surface tip B point while the other crossing point 4 is near the middle between B and Y points. This would give a CDW gap maximum close to B point and a gap minimum at Y point. This is also consistent with the observed CDW gap on the γ Fermi surface in Fig. 4. For the δ Fermi surface, the two crossing points 5 and 6 are close to the Fermi surface tip C point. This would give rise to a CDW gap maximum near C point and a gap minimum at Z point. This is also consistent with the measured result in Fig. 4. Overall, the observed CDW gap anisotropy is in a qualitative agreement with the expected results from the Fermi surface folding picture.

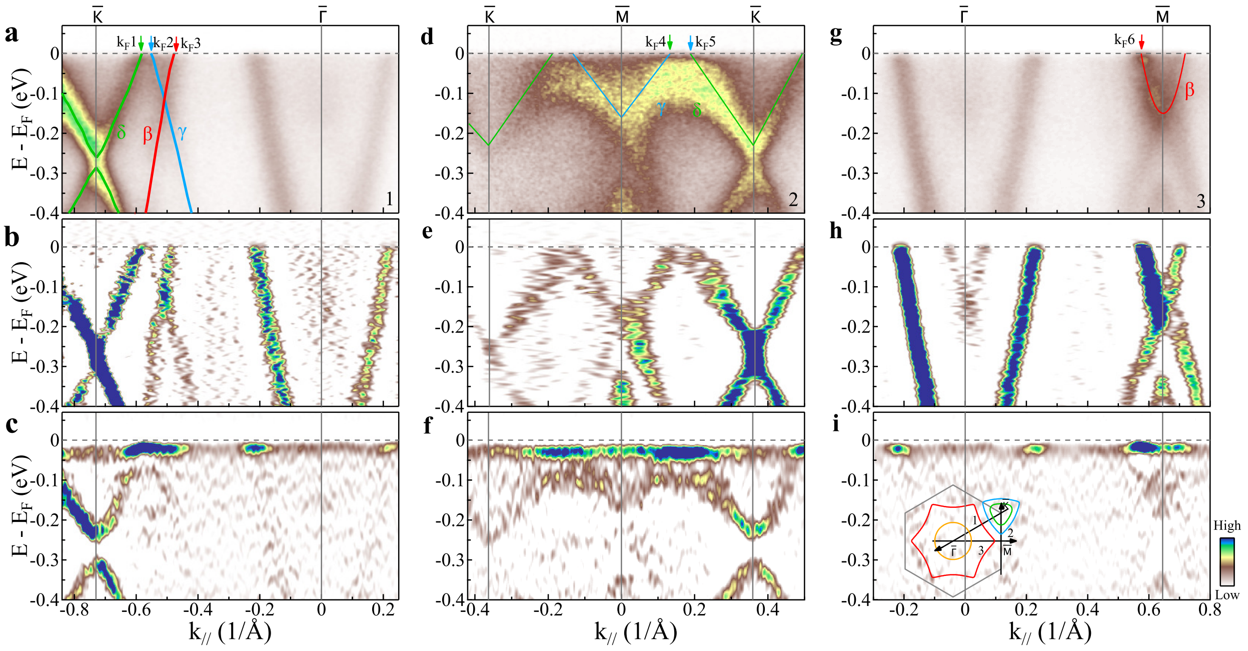

Supplementary Fig. 6: **Analysis of KV_3Sb_5 band structures measured along high-symmetry directions at 20 K.** (a) Band structure measured along the $\bar{K}$-$\bar{\Gamma}$ high-symmetry direction (cut 1). The location of the momentum cut 1 is shown in the inset of (i). (b) The second derivative image of (a) with respect to momentum. (c) The second derivative image of (a) with respect to energy. The combined analysis of (b) and (c) gives the measured bands shown as solid lines in (a). The Fermi momenta of the δ, γ and β bands are marked by arrows and labeled as $k_F1 \sim k_F3$. (d-f) The same as (a), (b) and (c) but measured along the $\bar{K}$-$\bar{M}$-$\bar{K}$ high-symmetry direction (cut 2). The observed bands are shown in (d) by solid lines. The Fermi momenta of the γ and δ bands are marked by arrows and labeled as k_F4 and k_F5. (g-i) The same as (a), (b) and (c) but measured along the $\bar{\Gamma}$-$\bar{M}$-$\bar{\Gamma}$ high-symmetry direction (cut 3). The observed band is shown in (g) by a solid curve. The Fermi momentum of the β band is marked by an arrow and labeled as k_F6.
